# P73 Improving antimicrobial stewardship on a paediatric intensive care unit at a large teaching hospital

**DOI:** 10.1093/jacamr/dlag102.079

**Published:** 2026-06-26

**Authors:** Kelly Atack, Lam Nguyen, Charlotte Fuller, Kavita Sethi, Fawaz Arshad

**Affiliations:** Leeds Teaching Hospitals, Leeds, UK; Leeds Teaching Hospitals, Leeds, UK; Leeds Teaching Hospitals, Leeds, UK; Leeds Teaching Hospitals, Leeds, UK; Leeds Teaching Hospitals, Leeds, UK

## Abstract

**Background:**

#CARES was implemented in Leeds Children’s Hospitals (LCH) as a mechanism to monitor the review of IV antimicrobials in September 2024. #CARES is a trust wide initiative that directs clinicians to document #CARES in the medical notes once they have completed an IV antimicrobial review. The acronym is based on the Start Smart then Focus guidelines and refers to the outcome of a review, including Cease, Amend, Refer, Extend and Switch. When #CARES is documented, robotic processing automation is utilized to count the reviews at ward, clinical service unit and trust wide level. #CARES is a trust wide key performance indicator, yet it has been challenging to implement due to the very large and diverse clinical services that Leeds Teaching Hospitals has, which create varying working practices. Leeds Children’s Hospital data for #CARES was lower than required, so the Paediatric Intensive Care Unit (PICU) was utilized as a pilot site to improve the rates of #CARES, with a view to providing measurable improvements to antimicrobial stewardship and using this as a showcasing opportunity to support the roll out further within the Children’s Hospital.

**Objectives:**

To increase the rate of #CARES documentation and therefore improve the quality of IV antimicrobial reviews on the Paediatric Intensive Care Unit. To review the impact of #CARES on prescribing of IV antimicrobials. To identify whether increased scrutiny on IV antimicrobial review had an impact on the rate of blood cultures being taken and sent for testing.

**Methods:**

An audit was undertaken pre-#CARES implementation. Promotion of #CARES was intensified on the unit, with reinforcement from infection specialists. A re-audit was undertaken to identify whether there were any changes.

**Results:**

26 treatment courses were examined in the first audit, with 67.8% of these having blood cultures sent, 80.8% of IV antimicrobials being reviewed at 48-72 h and only 19% of these having #CARES documented. In the first audit, 57.2 % of antibiotics were continued at the review and the average duration of the antibiotic course was 5.8 days. Post implementation, 29 treatment courses were reviewed. 77.8% of patients had blood cultures sent prior to the initiation of antibiotics. Review documentation improved, with 96.8% of patients having a review within 48–72 h and a significant increase in #CARES being documented at 75%. A reduction was seen in the proportion of antibiotics being continued, with 32.1% of antibiotics being continued at the 48–72 h. There was also a reduction in the duration of antibiotics, with the average being 3.7 days.

**Conclusions:**

Though a small sample, this project shows measurable improvements in stewardship on PICU, suggesting that utilizing #CARES can support reduction in the proportion of antibiotics continued at the 48-72 h review and the duration of antibiotics in particular. The success of this project will now be shared with the rest of the Children’s Hospital to support improvement in antimicrobial stewardship.

